# Supplementary material for: Vemurafenib Inhibits Acute and Chronic Enterovirus Infection by Affecting Cellular Kinase Phosphatidylinositol 4-Kinase Type IIIβ
Source: Microbiol Spectr. 2023 Jul 12;11(4):e00552-23. doi: 10.1128/spectrum.00552-23 (PMC10433971; doi:10.1128/spectrum.00552-23)
Supplement: Supplemental file 1 — Figures S1 and S2. Download spectrum.00552-23-s0001.docx, DOCX file, 0.6 MB [file spectrum.00552-23-s0001.docx]

**Vemurafenib inhibits acute and chronic enterovirus infection by affecting cellular kinase phosphatidylinositol 4-kinase type IIIβ**

**Supplementary material**

**Fig.S1**


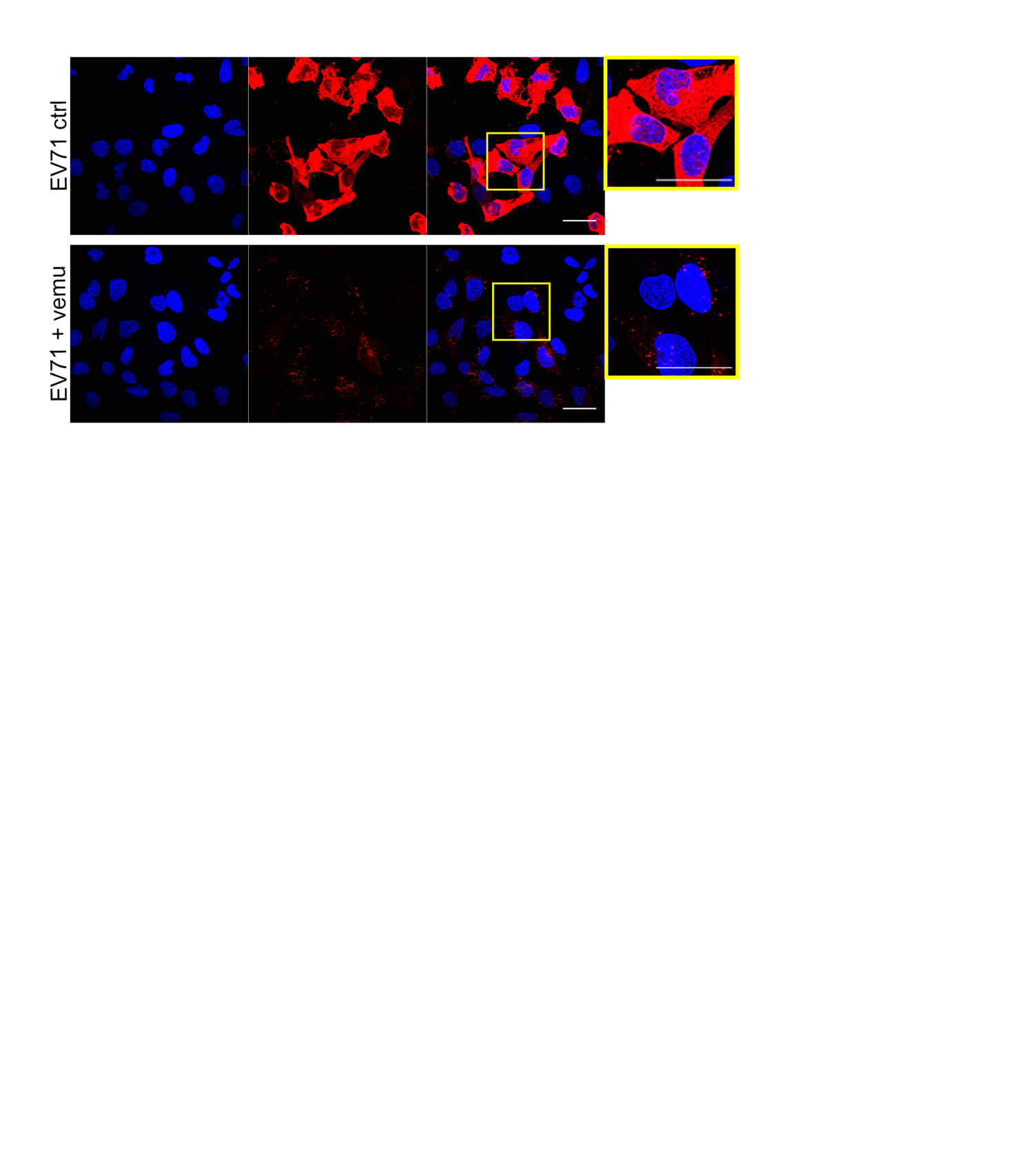


**Fig.S1. The protein production of EV71 is inhibited upon vemurafenib treatment.** RD cells (ATCC) were infected with EV71 (MOI 10) for 7.5 h in the presence of 5 µM vemurafenib diluted in DMEM supplemented with 1 % FBS and 1% glutamax. After fixation with 4 % PFA, the cells were immunolabeled with an antibody against EV71 (#MAB979, Merck) which is visible in red. Nuclei are visible in blue. Scale bars 40 µm.

**Fig.S2**





**FigS2.** Hela cells were infected with Renilla luciferase constructs RLuc-CVB3 WT, RLuc-CVB3-2C [AVIVAV] or RLuc-CVB3-3A [H57Y] for 30 min (MOI 0.1). Subsequently, the supernatant was replaced with DMEM containing the compound BF738735 or s-fluoxetine (SFX). Finally, the cells were lysed, and luciferase activity measured, or cell viability determined at 7 h p.i. Values are mean ±SD from three replicates and IC50 value was calculated using non-linear regression analysis.
